# The chemistry and histology of sexually dimorphic mental glands in the freshwater turtle, *Mauremys leprosa*

**DOI:** 10.7717/peerj.9047

**Published:** 2020-05-15

**Authors:** Alejandro Ibáñez, Albert Martínez-Silvestre, Dagmara Podkowa, Aneta Woźniakiewicz, Michał Woźniakiewicz, Maciej Pabijan

**Affiliations:** 1 Department of Comparative Anatomy, Institute of Zoology and Biomedical Research Jagiellonian University, Krakow, Poland; 2 Catalonian Reptile and Amphibian Rescue Centre-CRARC, Masquefa, Spain; 3 Department of Analytical Chemistry, Laboratory for Forensic Chemistry, Faculty of Chemistry, Jagiellonian University, Krakow, Poland

**Keywords:** Geoemydidae, Mental glands, GC-MS, Lipids, Secretions, Semiochemicals, TEM

## Abstract

Despite evidence from anatomy, behavior and genomics indicating that the sense of smell in turtles is important, our understanding of chemical communication in this group is still rudimentary. Our aim was to describe the microanatomy of mental glands (MGs) in a freshwater turtle, *Mauremys leprosa* (Geoemydidae), and to assess the chemical composition of their secretions with respect to variation among individuals and between sexes. MGs are paired sac-like organs on the gular region of the neck and are dimorphic in this species with males having fully functional holocrine glands while those of females appear non-secretory and vestigial. In adult males, the glandular epithelium of the inner portion of the gland provides exocytotic products as well as cellular debris into the lumen of the gland. The contents of the lumen can be secreted through the narrow duct portion of the gland ending in an orifice on the surface of the skin. Females have invaginated structures similar in general outline to male glands, but lack a glandular epithelium. Using gas chromatography coupled to mass spectrometry, we identified a total of 61 compounds in mental gland secretions, the most numerous being carboxylic acids, carbohydrates, alkanes, steroids and alcohols. The number of compounds per individual varied widely (mean (median) ± SD = 14.54 (13) ± 8.44; min = 3; max = 40), but only cholesterol was found in all samples. We found that the relative abundances of only six chemicals were different between the sexes, although males tended to have larger amounts of particular compounds. Although the lipid fraction of mental gland secretions is rich in chemical compounds, most occur in both sexes suggesting that they are metabolic byproducts with no role in chemical signaling. However, the relative amounts of some compounds tended to be higher in males, with significantly larger amounts of two carboxylic acids and one steroid, suggesting their putative involvement in chemical communication.

## Introduction

Animals communicate with other members of their species in a wide variety of contexts over their lifetimes. Communication is essential for many crucial activities, as for example, avoidance of aggressive encounters among conspecifics in territorial species and/or choosing mating partners for reproduction. Given the relevance of the process, animals have developed the ability to exploit several signaling channels or pathways to transmit pertinent information, with acoustic, chemical, tactile and visual signals being among the most common.

Chemical signals have been extensively studied in certain groups of invertebrates such as insects (Symonds & Elgar, 2008). Chemical communication in vertebrates is less understood; however, a relatively large array of molecules has been described in a limited set of taxa, indicating an underlying complexity of chemical signaling in this group (Weldon, Flachsbarth & Schulz, 2008; Wyatt, 2014; Apps, Weldon & Kramer, 2015). In some cases—especially in mammals—the chemical compounds have been isolated and their functions are known. For instance, major urinary proteins are involved in individual identity in wild house mice *Mus domesticus* (Hurst et al., 2001). However, for most vertebrates chemical compounds with a (potential) signaling role have not been identified. Reptilian chemosignals have been studied in a taxonomically restricted set of species (Martín & López, 2011). Femoral glands in lacertid and iguanid lizards and the skin of colubrid snakes are known to secrete sex-specific chemosignals (Weldon, Flachsbarth & Schulz, 2008; Houck, 2009). For example, skin-borne methyl ketones released by female red-sided garter snakes (*Thamnophis sirtalis parietalis*) are used as male attractants and might convey information on female size and indicate fecundity (Lemaster & Mason, 2002). Furthermore, the variation in methyl ketone composition between different species within the genus *Thamnophis* is species-specific and thus could be a driver of speciation (Uhrig, LeMaster & Mason, 2014). Other compounds such as vitamin E might be reliable signals of male quality in lizards (Martín & López, 2010; García-Roa et al., 2017; Kopena, López & Martín, 2017). These results indicate that assessing the level of inter-individual and sex-specific variation in chemical signals is an essential step in understanding the functionality and evolution of semiochemical compounds in vertebrates.

Several lines of evidence indicate that chelonians have a well-developed olfactory system. First, the draft genomes of soft-shell (*Pelodiscus sinensis*) and green sea (*Chelonia mydas*) turtles have revealed extensive and independent expansion of functional olfactory receptor genes (Wang et al., 2013). Second, a number of behavioral studies have provided indirect evidence for the importance of olfactory cues in intra and intersexual recognition (Rose, 1970; Weaver, 1970; Muñoz, 2004; Poschadel, Meyer-Lucht & Plath, 2006; Lewis et al., 2007; Mason & Parker, 2010; Ibáñez, López & Martín, 2012). For instance, freshwater turtles might detect chemicals from other conspecifics that can be relevant to mate finding or for establishing dominance between members of their own species (Poschadel, Meyer-Lucht & Plath, 2006; Ibáñez, López & Martín, 2012; Ibáñez et al., 2013). Third, some turtle species have specialized secretory organs such as cloacal glands and/or Rathke’s glands in the inguinal or axillary regions (Waagen, 1972; Ehrenfeld & Ehrenfeld, 1973; Mason & Parker, 2010). In addition, numerous chelonian species within the superfamily Testudinoidea possess mental glands (MGs), also called subdentary or chin glands, located in the integument of the gular part of the neck (Winokur & Legler, 1975). A fourth line of evidence comes from chemical analysis of the contents of MGs in desert-dwelling gopher tortoises (*Gopherus*) in which these glands are particularly pronounced (Rose, Drotman & Weaver, 1969; Rose, 1970; Weaver, 1970; Alberts, Rostal & Lance, 1994). Functionally, it has been demonstrated that secretions produced by MGs might mediate sex recognition and male interactions in *G. berlandieri* and *G. agassizii* (Rose, 1970; Weaver, 1970; Alberts, Rostal & Lance, 1994). Chemical analysis revealed the presence of proteins of varying molecular size as well as lipid compounds such as cholesterol, phospholipids, triglycerides and free fatty acids (Rose, Drotman & Weaver, 1969; Rose, 1970; Alberts, Rostal & Lance, 1994). Overall, the available data indicates that chemical communication in chelonians is widespread and occurs through the production of chemical compounds in different types of secreting organs. However, mental gland microanatomy has only been assessed superficially using standard light microscopy, while the ultrastructure of these organs remains unknown (Rose, Drotman & Weaver, 1969; Weaver, 1970; Winokur & Legler, 1975). Furthermore, with the exception of electrophoretic and chromatographic studies on secretions in *Gopherus* (Rose, Drotman & Weaver, 1969; Rose, 1970; Alberts, Rostal & Lance, 1994), nearly nothing is known of the chemical compounds produced by MG secretions.

In this article we report the chemical composition and microanatomy of the MGs of the Spanish terrapin, *M. leprosa* (Geoemydidae, former Bataguridae), a sexually dimorphic and predominantly freshwater species distributed in northwestern Africa and the Iberian peninsula into southern France (Díaz-Paniagua, Andreu & Keller, 2015; Bertolero & Busack, 2017). Our aims are to (i) characterize the chemical composition of MG secretions in this species, and (ii) test if there are sex-specific differences in MG gland components and gland structure in *M. leprosa*, known for using chemical cues to discriminate among potential partners and avoid competitors (Ibáñez, López & Martín, 2012; Ibáñez et al., 2013). We confront the chemical profiles of the freshwater *M. leprosa* MGs with those of *Gopherus* spp. inhabiting xeric environments. In addition, under the assumption that MG secretions play a role in the reproduction of this species, we expect that male MGs are structurally more complex than female glands, and that they produce sex-specific compounds absent in the latter. We use gas chromatography coupled to mass spectrometry (GC–MS) to characterize MG chemical compositions in both sexes of this species and apply a combination of light microscopy (LM) and transmission electron microscopy (TEM) to provide a detailed assessment of the structure of these glands.

## Materials and Methods

### Study animals

The individuals of *M. leprosa* examined in this study came from wild Iberian populations and were housed outdoors in semi-natural conditions in the facilities of the Catalonian Reptile and Amphibian Rescue Center (CRARC). Turtles were fed regularly and were part of a reproductive program aimed at releasing individuals into wetlands that are recovering ecologically. The turtles’ diet included fish, crabs, algae and aquatic plants, at times supplemented by chicken, fish and lettuce from the market. All individuals used in this study were adults sexed according to external morphological features.

The CRARC holds Catalan permit B2100126 for Zoo Facilities to maintain reptiles, including *M. leprosa*, in captivity. The sampling of MGs was accepted by the institutional board of CRARC. Following European Union directive 2010/63/UE, the extraction of mental gland exudates does not qualify as an experimental procedure because it does not puncture the tissue or harm the turtle. The sampling protocol was performed following standard rules of animal welfare and certification CPISR-1 C29052019 granted by the Departament de Territori i Sostenibilitat, Generalitat de Catalunya (Spain) to Albert Martínez-Silvestre.

### Dissection and histology of mental glands

Mental glands from four adult turtle specimens (three males and one female) from CRARC were used for histological examination. MGs were dissected from freshly dead turtles and stored in chemical buffers (see below). Turtles died from trauma-related injuries and the necropsy procedure was performed at CRARC by a specialized veterinarian (A Martínez-Silvestre). MG structure was examined using LM and TEM.

For examination in LM, entire MGs were fixed in Bouin’s solution immediately after excision. Dehydration was done in an alcohol gradient followed by clearing in xylene and embedding in paraffin as previously described (Piprek et al., 2012). Sectioning of paraffin blocks was conducted on a ZEISS HYRAX microtome. Paraffin sections (6 μm thick) were stained with Harris’s hematoxylin and eosin to visualize the general structure of the glands. Alcian blue staining was used to detect acidic polysaccharides. Periodic acid-Schiff (PAS) stain was used to detect polysaccharides. Mallory’s trichrome stain was used for the visualization of collagen (Kiernan, 1990).

Small fragments of MGs used for TEM were fixed in Karnovsky’s fixative (Karnowsky, 1965). The material was washed in 0.1 M cacodylate buffer and post-fixed in 1% osmium tetroxide. Dehydration was carried out in a series of graded ethanol solutions. Then the material was embedded in epoxy resin (EPON-812) as previously described (Piprek et al., 2017). Semithin sections (0.5 μm) of the resin were stained with methylene blue and Azure II (1:1) for LM examination. Ultrathin sections (60–70 nm) were contrasted with uranyl acetate and lead citrate for TEM examination. Images were collected with a JEOL-2100HT transmission electron microscope in the Department of Cell Biology and Imaging, Jagiellonian University (Kraków, Poland).

LM and TEM images were processed in CorelDRAW and Corel Photo-Paint to create the layout of the figures. Basic adjustments on image brightness, contrast, intensity and tone were performed if needed.

### Collection of mental gland secretions for chemical analysis

We sampled live turtles of both sexes in two different years or seasons (August–September 2018 and March 2019) at CRARC. Secretions from a total of 38 individuals were sampled and analyzed using GC–MS but only 37 (21 males and 16 females) were included in the statistical analysis (see below). Samples were refrigerated after sample collection and stored in cold conditions (−20 °C) until chemical analysis. We additionally collected blank control samples (opened and handled the same way as other samples but without taking secretion), as well as samples of water from the turtle enclosures to check for potential contaminants.

The procedure for MG sampling of live turtles (applicable to all small- and medium-sized chelonians possessing MGs) is outlined below. First, the head should be pulled out from inside the shell and immobilized. Second, the mouth should be carefully opened by using an oral avian speculum to pry apart the jaws. Third, mechanical pressure needs to be applied to the MGs from within the oral cavity by using forceps with curved tips. Usually secretions are then released from the orifices of the glands located in the gular region, but mechanical pressure can also be applied at the margins of the gland with forceps to squeeze the glands. The duration of the procedure was less than 10 min per turtle. Secretions can be gathered directly into collection vials, but usually they are collected using forceps if in solid state, or pipetted by glass microcapillary tubes if in liquid state and then deposited in glass vials. Forceps and other tools used for the collection of secretions should be cleaned with dichloromethane before the sampling process to minimize contamination. Glass microcapillary tubes should be used only once, then disposed of. MG exudates were collected in glass vials previously filled with dichloromethane and closed with silicone/PTFE screw caps.

### Chemical analysis

Before the GC–MS analysis, all samples were subjected to a derivatization protocol to introduce a trimethylsilyl functional group to the compound of interest. First, samples were warmed up to ambient temperature and dichloromethane was evaporated to dryness under a stream of nitrogen at 40 °C. Then, 10 μL of acetonitrile and 50 μL of 99% N,O-bis(trimethylsilyl)trifluoroacetamide (BSTFA) with 1% trimethylchlorosilane (TMCS) mixture were added into the dry residue. The amber glass vials were tightly closed and the derivatization process was carried out at 60 °C for 1 h. Afterwards, the vials were opened and the derivatization solution was evaporated under a stream of nitrogen at 60 °C and the dry residue was dissolved in 25 μL of dichloromethane. Water from the pond was extracted using dichloromethane and ethyl acetate. Briefly, extracting solvent and water samples were mixed in a ratio of 2:1, respectively shaken for 10 min, then phases were separated by centrifugation and the organic layer was collected. Three consecutive extractions were performed for every sample, and the separated organic solvents were put into one vial (separate for each solvent type). The collected solvent was evaporated to dryness under a stream of nitrogen at 40 °C and the dry residue was subjected to the derivatization procedure described above.

All samples were analyzed by a GC–MS system consisting of a 6850 Series II gas chromatograph and a 5975C MSD mass spectrometer (Agilent Technologies, Santa Clara, CA, USA) equipped with a HP-5ms capillary column (30 m long, 0.25 mm i.d., 0.25 μm film thickness, Agilent Technologies, Santa Clara, CA, USA). The oven temperature program was set up to hold 50 °C for 10 min, then ramp the temperature up to 280 °C with a rate of 5 °C/min, and then hold for 30 min. Helium (5.0) was used as a carrier gas with a flow rate of 1.0 mL/min. Splitless injection of two μL was performed at injection port heated to 280 °C. The MS transfer line temperature was set to 280 °C and ion source temperature to 230 °C. EI source operated at 70 eV, and the mass range for the MS detector in scan mode was from 39 to 400 m/z (from 5 min) and from 39 to 600 m/z (from 20 min). The identification of all detected compounds was based on a semi-automatic library search (all results were inspected by the operator) with the NIST 11 database (NIST, Gaithersburg, MA, USA).

### Data filtering and statistical analysis

Compounds were tentatively identified on the basis of their mass spectra match and the retention times of detected peaks were additionally used to compare samples. First, we excluded unmatched compounds as well as compounds with a match lower than 850, considering them as unidentified. Afterwards, we constructed a database with all the identified compounds and filtered out compounds that appeared only in one sample (including potential contaminants). Thus, only compounds appearing in at least two samples were retained. Likewise, substances occurring in two or more control tubes were considered as contaminants and excluded from the downstream analysis. In several instances, compounds considered here as contaminants were non-natural products (e.g., phthalates). In a few cases compounds that could naturally appear in MG secretions were excluded as they were found in at least two control samples. After all filtration steps, one of the samples contained only cholesterol trimethylsilyl ether and was therefore excluded from the statistical analysis. In several cases some non-derivatized parent compounds were found present next to their derivatives. In such cases, only trimethylsilyl derivatives were taken into account for statistical analysis, and thus, non-derived compounds were excluded. Substances resistant to derivatization (sylilation process), for example, alkanes, were considered in their parent form.

To calculate the relative amounts of the compounds, we used the ratio of the area of an identified compound divided by the area of a compound present in all samples. The contaminant: phthalic acid, hept-4-yl isobutyl ester was chosen as a compound likely originating from the dichloromethane bottle sealing used during sampling and it was present in all samples. The ratios of the compounds were used for further analysis.

Potential sexual differences were tested using ANOSIM on the ratio matrix. A distance matrix (*vegdist* function, Bray–Curtis option) was calculated using the ratio matrix as input. The distance matrix was used for non-metric multidimensional scaling (NMDS) plots to visualize inter-individual and sexual variation in chemical composition. NMDS plots were done using the function metaMDS in the vegan package (Oksanen et al., 2019). Cholesterol trimethylsilyl ether was found in all samples, but its relative amount varied greatly among samples. This compound was excluded from some analyses to avoid its potentially confounding effect (see “Results”). Lipid profiles were examined in more detail to explore sexual differences in specific compounds. Only alcohols, alkanes, carboxylic acids and steroids (excluding cholesterol) were selected for this analysis (carbohydrates and other classes were not included). Differences between the sexes in the amounts of selected compounds were assessed with a Wilcoxon rank sum test with continuity correction using the R-function *wilcox.test*. The *p* values were adjusted for multiple comparisons using the R function *p*.adjust (method = “fdr”) (Benjamini & Hochberg, 1995). Temporal variation in MG chemistry was explored only for males due to the higher number of available samples.

The method used here, that is, the use of an internal standard to calculate the relative amounts of the compounds, has the advantage of not being affected by the number of peaks present in the sample. However, as we did not measure or weigh the secretions, it is likely that different amounts of secretions were collected for each individual. Therefore we also calculated the relative amounts of the compounds by another commonly used method. The percentage of each compound was calculated as the area of the compound divided by the total area (summation) of the rest of the identified compounds (i.e., area of the compound divided by the total area of the identified compounds in the sample). We used percentages to visualize sexual differences. A similar pattern was observed with percentages (see NMDS plot; Fig. S1) compared to the relative amounts calculated with the internal standard and therefore no further analyses using percentages were carried out.

Analysis and some plots were performed in R version 3.4.4 (R Core Team, 2018) using the interface Rstudio. Chemical profiles were visualized using the software Instant Clue (Nolte et al., 2018).

## Results

### General structure of mental glands in males

**Figure 1:**
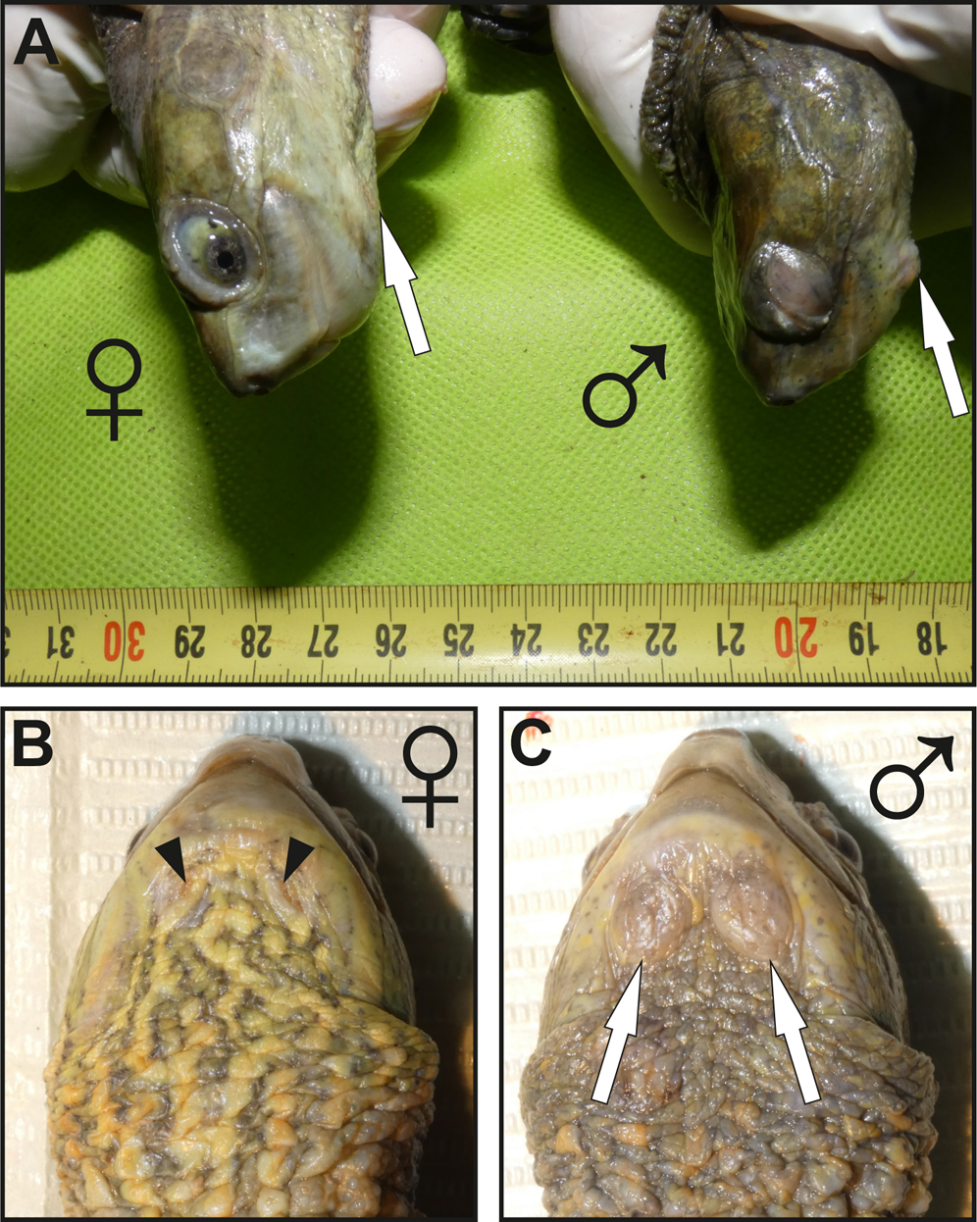
Images showing the macroscopic aspect of mental glands in *Mauremys leprosa*. (A) Lateral view of the head in female (♀) and male (♂); more prominent mental glands (arrows) are noticeable in the male. (B and C) Ventral view of the gular region of a female (♀) (B) and male (♂) (C), the orifices (openings) of the glands are clearly visible in females and are filled by brownish plugs (arrowheads), unlike males in which orifices are not easily visible. Specimens pictured in (A), (B) and (C) are not the same.

**Figure 2:**
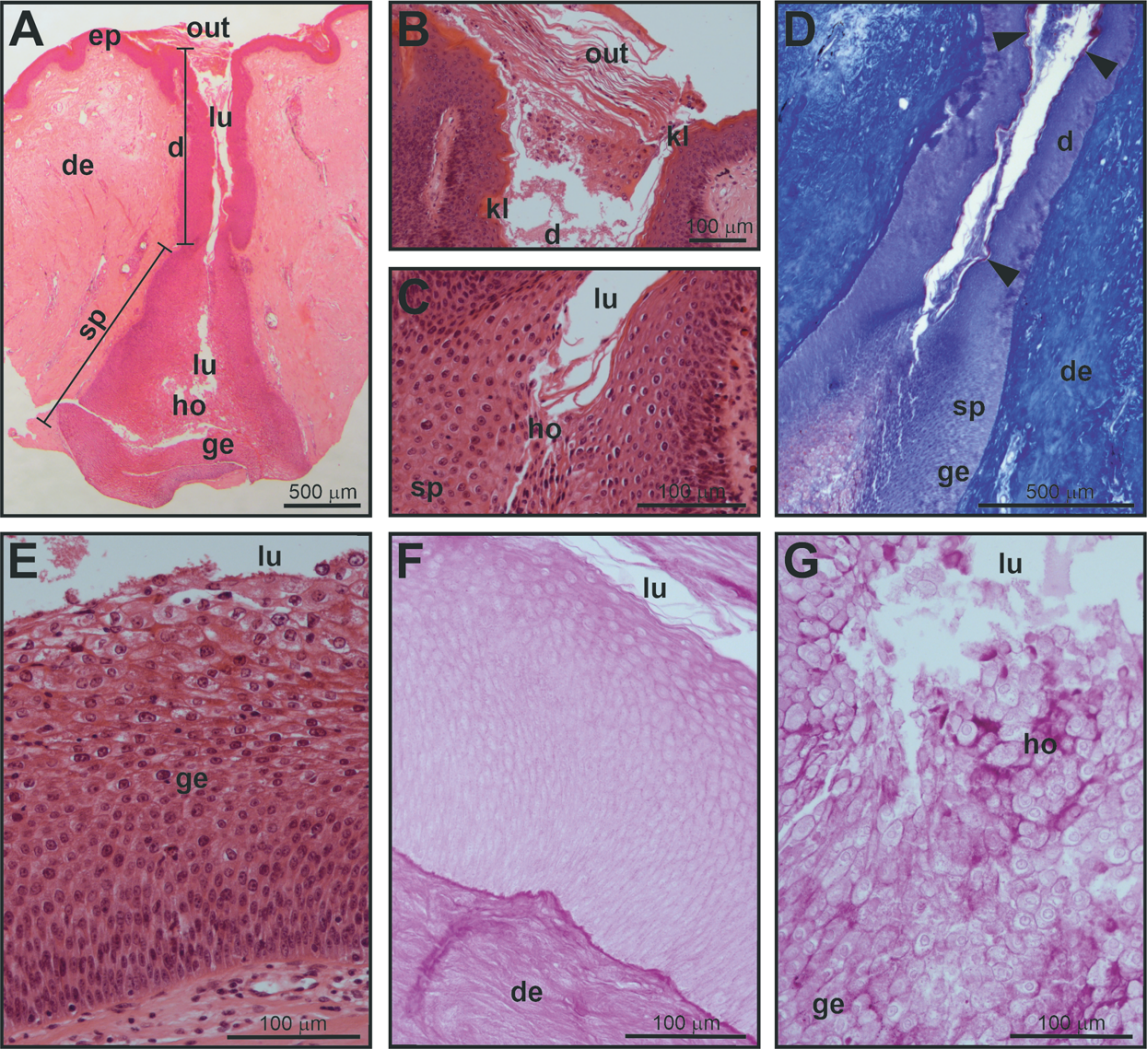
Histological structure of a mental gland in a male of *M. leprosa*. (A) Section of the full mental gland, hematoxylin-eosin (HE) staining (scale is approximate). (B) Detail of the outlet (opening), the outlet is plugged with secretion and exfoliated keratinized epithelium, HE staining. (C) Detail of the transition between the excretory duct and the secretory portion, HE staining. (D) Detail of the excretory duct and secretory portion, Mallory’s Trichrome; note the presence of a keratinized layer in the excretory duct stained red (arrowheads); connective tissue in the dermis is stained blue. (E) Detail of the glandular epithelium and the secretory portion (see Fig. 3 for detail on the fine structure of the different cell layers in the glandular epithelium), HE staining. (F) PAS staining in the excretory duct of the mental gland. (G) Positive PAS staining (intense purple color) of the holocrine secretion in the secretory portion of the mental gland. d, excretory duct; de, dermis; ep, epidermis; ge, glandular epithelium; ho, holocrine secretion; kl, keratinized layer; lu, lumen; out, plugged outlet of the gland; sp, secretory portion.

In external morphology, MGs in males consist of two bulges located on both sides of the gular region (ventral surface of the neck) and are relatively prominent (Fig. 1A). A pair of orifices (openings), leading to the MGs are present on each side of the neck. Orifices are hardly visible to the naked eye in males (see Figs. 1B and 1C for a comparison with females, in which openings are more visible). LM showed that each of these four external orifices leads to sac-shaped elongated epidermal invaginations (Fig. 2A). There is no evidence from our histological examination that these invaginations are interconnected, therefore they are likely separate units, that is, each turtle possesses four separate exocrine MGs with separate openings (two on each side of the neck). The histological structure of each gland is similar, although one of each pair is slightly larger than the other. MGs are sac-shaped, simple acinar glands consisting of two parts: (1) an excretory duct opening with an outlet (orifice) at the skin surface, and (2) an inner-most secretory portion (Fig. 2A). The outlet and the excretory duct are lined by a keratin layer (*stratum corneum*) which is continuous with the outer keratinized layer of the epidermis at the surface of the neck (Figs. 2A and 2B). The excretory duct is narrow and possesses a lumen inside (Figs. 2A–2D). The gland outlet is plugged with secretion and exfoliated keratinized epithelium (Fig. 2B). The excretory duct, as a narrow tube, opens into the lumen of the wide, sac-shaped basal part of the gland (Figs. 2A and 2D). At the interface between the excretory duct and secretory portion (Fig. 2C), the keratinized layer becomes thin, ultimately disappearing, and is replaced by glandular epithelium in the secretory portion. The secretory portion is lined exclusively with thick, multilayer glandular epithelium (Fig. 2E). The lumen of the secretory portion is filled with a mix of the contents of glandular cells and their derived products, demonstrating that MGs are holocrine (Fig. 2E). When MG tissue was tested with PAS (Figs. 2F and 2G), the holocrine secretion showed a weak positive reaction suggesting that carbohydrates or polysaccharides are likely present (Fig. 2G). MG tissue did not stain with alcian blue, indicating a lack of acidic polysaccharides.

**Figure 3:**
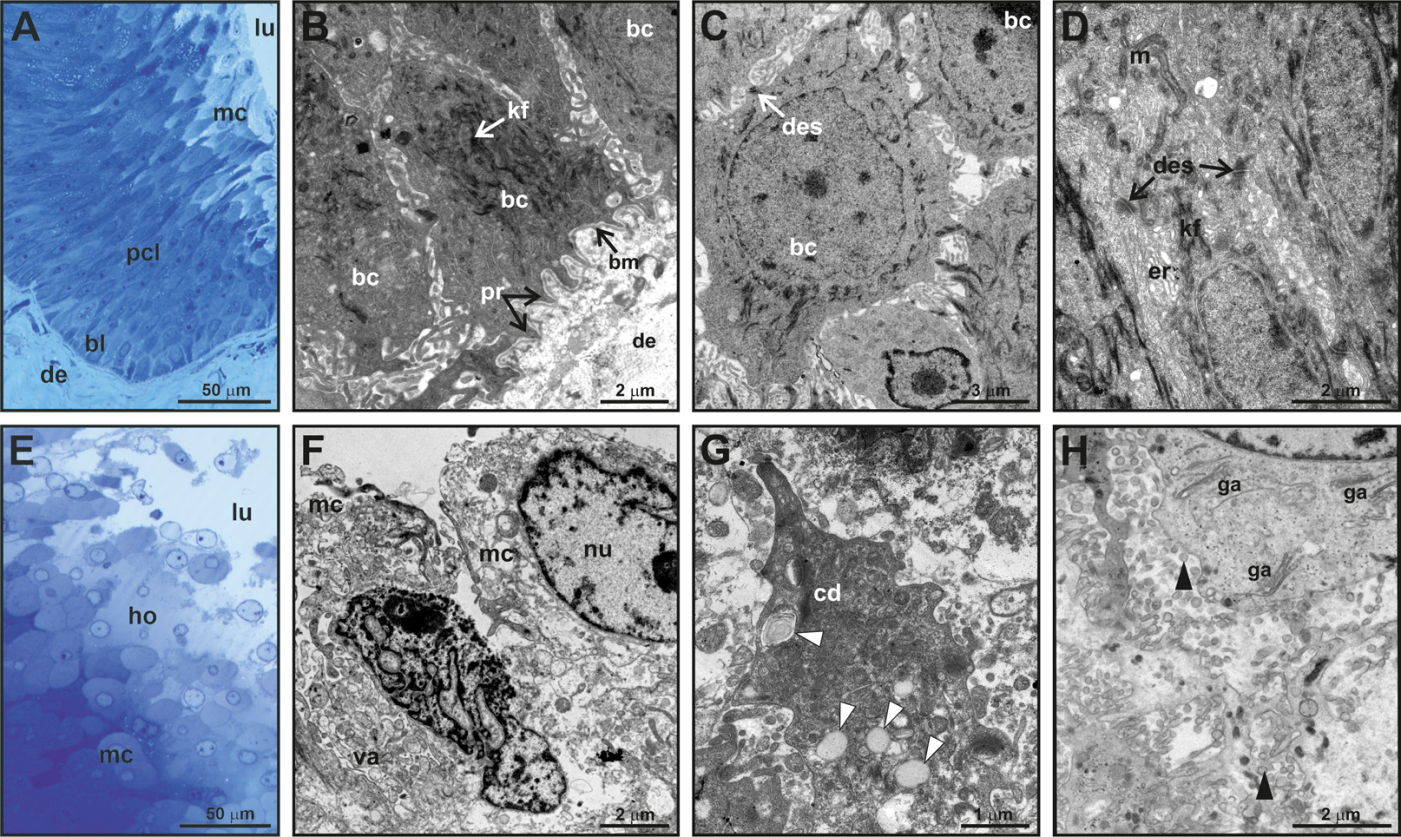
Fine structure of the glandular epithelium of mental glands of *M. leprosa* males. (A) Semithin section showing the different layers of the glandular epidermis, methylene blue–Azure II. (B) Section (TEM) of the basal layer showing basal cells with protrusions invaginating through the connective tissue. (C) Detail (TEM) of basal cells. (D) Section (TEM) of polyhedral cells constituting the prickled cell layer. (E) Semithin section of the mature cells and holocrine secretion, methylene blue-azure II. (F) Mature cells (TEM) that disintegrate in the lumen with abundant vacuoles. (G) Detail of cytoplasm fragments and electron-light bodies (white arrowheads). (H) Mature cell (TEM) in the lumen of the gland. Note the presence of Golgi apparatus, exocytotic vesicles (black arrowheads), free electron-dense bodies and fragments of cell membranes in the lumen of the gland. bl, basal cell layer; bc, basal cell; bm, basal membrane; cd, cytoplasmic discharge; des, desmosome; lu, lumen; er, endoplasmic reticulum; ga, Golgi apparatus; kf, keratin fibers; ho, holocrine secretion; pcl, prickled cell layer; nu, nucleus; m, mitochondria; mc, mature cell; pr, protusion; va, vacuoles.

Fine scale examination of MG tissue in TEM showed that the glandular epithelium comprises an engrossed basal layer of epithelium (*stratum basale*) consisting of dividing cells that successively differentiate toward the next intermediate cell layer (similar to the prickle cell layer, that is, *stratum spinosum* in normal skin epidermis) until maturing and disintegrating into the glandular lumen (Fig. 3). TEM observations revealed that the inner-most layer of the glandular epithelium (i.e., basal layer) contains basal cells that have thick bundles of keratin as well as a nucleus rich in chromatin (Figs. 3B and 3C). Basal cells are anchored to the dermis by numerous cytoplasmic protrusions that fold into the basement membrane (Fig. 3B). The *stratum spinosum* possesses polyhedral cells that are more elongated than basal cells (Figs. 3A and 3D). Prickle cells are loosely arranged, and there are intercellular spaces between their cytoplasmic projections. Toward the lumen, cells from this intermediate layer are more tightly packed, and their cytoplasms form protrusions connected by desmosomes (Fig. 3D). These cells are rich in mitochondria. Close to the lumen of the gland, cells differentiate into mature holocrine cells that are swollen and irregular in shape (Figs. 3E and 3F). Cells disintegrate, nuclei become fragmented and the cytoplasm together with other cell products is discharged into the lumen (Figs. 3E–3H). The holocrine secretions are therefore composed of the excretion products of the cell as well as organellular debris and cellular membrane fragments (Fig. 3G). Electron-light bodies and small electron-dense bodies were also detected in the lumen of the gland (Figs. 3G and 3H). Mature cells have abundant Golgi apparatus that probably play a key role in exocytosis occurring in mature cells (Fig. 3H).

### General structure of mental glands in females

**Figure 4:**
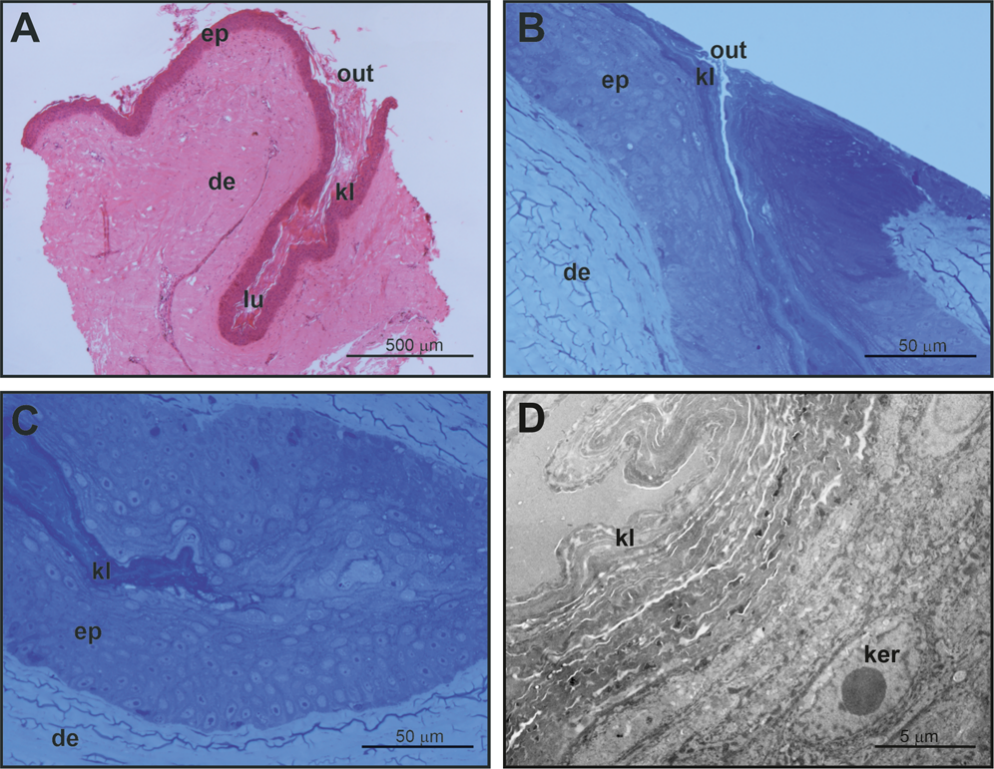
Histological and fine structure of mental glands in a female of *M. leprosa*. (A) Lateral section stained with HE. (B) Superficial region of the gland (semithin section) (C) Detail of the basal part of the gland (semithin section). (D) Basal part of the gland (TEM). de, dermis; ep, epidermis; ker, keratinocyte; kl, keratin layer; lu, lumen; out, outlet of the gland.

Mental glands of females are much more reduced and less prominent compared to male glands (roughly one third of the length of male glands based on a single specimen, see Figs. 1, 2A and 4A for a rough comparison). Moreover, histological examination of female MGs also showed a lower degree of complexity (Fig. 4A). The female MG is a simple, short invagination of the epidermis without a wide secretory portion (Figs. 4B–4D). The entire female MG is lined with a keratinized epidermis similar to the epithelium of the excretory duct in the male MG. Thus, female MGs more closely resemble the regular skin epidermis and are notably different than those of males (Figs. 4C and 4D). As there was no evidence of secretion in females, PAS reaction was not carried out.

### Characterization of chemical compounds in mental glands

After filtration steps (see “Material and Methods”) a total of 61 chemical compounds were identified, at least to class, in the MG secretions of *M. leprosa* (Table 1). The number of compounds per individual was highly variable (mean (median) ± SD = 14.54 (13) ± 8.44; min = 3; max = 40). The most common compound was cholesterol trimethylsilyl ether (averaged relative area: 17.79). Although cholesterol trimethylsilyl ether was the only compound found in all samples, the amount per sample was extremely variable, ranging from a maximum of 105.6 to a minimum of 1.63. Besides cholesterol, the most abundant compounds were 5α-cholestan-3β-ol trimethylsilyl derivative (averaged relative area = 0.99), 3-((trimethylsilyl)oxy)lanosta-9(11),24-diene (0.66), carbohydrate unidentified 8 trimethylsilyl derivative (0.51) and campesterol trimethylsilyl ether (0.43) (see Table 1).

**Table 1:** Identified chemical compounds in *Mauremys leprosa* mental glands from the GC-MS. Relative amounts (Ratios; Mean and SD) of the compounds, calculated by using an internal standard (phthalic acid, hept-4-yl isobutyl ester), a non-natural compound appearing in all samples (see details in “Materials and Methods”). Relative amounts for males (mean) and females (mean), together with adjusted significance value for multiple comparisons (*P* Adj.) are shown—only alcohols, alkanes, carboxylic acids and steroids (except cholesterol trimethylsilyl ether) were considered in this analysis. Significant differences after adjustment are highlighted in bold. Abbreviations (Abrr.) as in Fig. 6. The number of males and females in which the compound was detected is provided in columns “M (num.)” and “F (num.)”. Percentage (mean) for each compound was calculated based on the area of the focal compound in respect to the total area of identified compounds.

Name	Abbr.	Ratios (mean)	SD	M (mean)	F (mean)	P Adj.	F (num.)	M (num.)	Percentage (mean)
**Alcohols**									
1-Hexadecanol, trimethylsilyl ether	Aol-1	0.046	0.207	0.075	0.007	0.817	1	2	0.112
Octadec-9Z-enol, trimethylsilyl ether derivative	Aol-2	0.022	0.082	0.039	0.000	0.250	0	4	0.061
1-O-Hexadecylglycerol, - bis(trimethylsilyl) ether derivative	Aol-3	0.109	0.200	0.114	0.102	0.907	7	10	0.350
1-O-Octadecylglycerol, - bis(trimethylsilyl) ether derivative	Aol-4	0.021	0.072	0.022	0.019	0.907	2	2	0.049
**Alkanes**									
Tricosane	Alk-1	0.019	0.085	0.026	0.010	0.907	2	2	0.053
Tetracosane	Alk-2	0.045	0.135	0.055	0.031	0.891	4	4	0.144
Pentacosane	Alk-3	0.055	0.183	0.057	0.052	0.350	5	2	0.187
Hexacosane	Alk-4	0.060	0.244	0.069	0.049	0.285	4	1	0.170
Heptacosane	Alk-5	0.066	0.238	0.077	0.052	0.483	4	2	0.183
Octacosane	Alk-6	0.060	0.220	0.071	0.047	0.624	3	2	0.169
Nonacosane	Alk-7	0.057	0.222	0.070	0.040	0.624	3	2	0.155
Triacontane	Alk-8	0.041	0.185	0.050	0.029	0.907	1	1	0.092
**Amines**									
1-Dodecanamine, N,N-dimethyl-	–	0.073	0.226	–	–	–	5	2	0.379
1-Tetradecanamine, N,N-dimethyl-	–	0.020	0.081	–	–	–	3	1	0.095
**Carbohydrates**									
Carbohydrate Unidentified 1, trimetylsilyl derivative	–	0.004	0.019	–	–	–	1	1	0.012
Carbohydrate Unidentified 2, trimetylsilyl derivative	–	0.009	0.035	–	–	–	3	1	0.031
Carbohydrate Unidentified 3, trimetylsilyl derivative	–	0.002	0.008	–	–	–	1	1	0.003
Carbohydrate Unidentified 5, trimetylsilyl derivative	–	0.101	0.286	–	–	–	1	7	0.789
Carbohydrate Unidentified 8, trimethylsilyl derivative	–	0.509	0.656	–	–	–	11	18	3.596
Carbohydrate Unidentified 9, trimethylsilyl derivative	–	0.213	0.321	–	–	–	4	14	1.827
Carbohydrate Unidentified 10, trimethylsilyl derivative	–	0.006	0.024	–	–	–	0	3	0.083
Carbohydrate Unidentified 11, trimethylsilyl derivative	–	0.096	0.318	–	–	–	0	4	0.450
Carbohydrate Unidentified 12, trimethylsilyl derivative	–	0.037	0.107	–	–	–	0	6	0.219
Carbohydrate Unidentified 13, trimethylsilyl derivative	–	0.104	0.263	–	–	–	0	8	0.697
Carbohydrate Unidentified 14, trimethylsilyl derivative	–	0.127	0.386	–	–	–	0	4	0.469
Carbohydrate Unidentified 6, trimetylsilyl derivative	–	0.286	0.394	–	–	–	11	16	1.883
Carbohydrate Unidentified 7, trimetylsilyl derivative	–	0.012	0.037	–	–	–	5	4	0.061
**Carboxylic acids**									
Propanoic acid, 2-[(trimethylsilyl)oxy]-, trimethylsilyl ester	Cac-1	0.055	0.331	0.098	0.000	0.420	0	2	0.191
Benzoic acid, trimethylsilyl ester	Cac-2	0.010	0.024	0.014	0.004	0.591	3	6	0.040
Nonanoic acid, trimethylsilyl ester	Cac-3	0.006	0.021	0.004	0.007	0.591	2	1	0.057
Dodecanoic acid, trimethylsilyl ester	Cac-4	0.020	0.054	0.034	0.001	0.197	1	7	0.052
Azelaic acid, bis(trimethylsilyl) ester	Cac-5	0.015	0.059	0.021	0.006	0.817	1	2	0.046
Tetradecanoic acid, trimethylsilyl ester	Cac-6	0.312	0.624	0.519	0.040	**0.028**	2	14	0.972
Pentadecanoic acid, trimethylsilyl ester. Isomer 1	Cac-7	0.021	0.068	0.036	0.002	0.420	1	4	0.048
Pentadecanoic acid, trimethylsilyl ester. Isomer 2	Cac-8	0.039	0.188	0.067	0.003	0.591	1	3	0.063
Pentadecanoic acid, trimethylsilyl ester. Isomer 3	Cac-9	0.015	0.064	0.027	0.000	0.420	0	2	0.023
Hexadecenoic acid, trimethylsilyl ester. Isomer 1	Cac-10	0.051	0.112	0.089	0.000	**0.037**	0	9	0.190
Hexadecenoic acid, trimethylsilyl ester. Isomer 2	Cac-11	0.329	0.734	0.515	0.085	0.285	7	12	0.953
Heptadecanoic acid, trimethylsilyl ester. Isomer 1	Cac-12	0.007	0.029	0.012	0.000	0.420	0	2	0.010
Heptadecenoic acid, trimethylsilyl ester	Cac-13	0.010	0.047	0.018	0.000	0.420	0	2	0.029
Heptadecanoic acid, trimethylsilyl ester. Isomer 2	Cac-14	0.042	0.101	0.068	0.007	0.285	2	7	0.155
Octadecadienoic acid, trimethylsilyl ester	Cac-15	0.329	0.641	0.488	0.120	0.247	5	12	1.033
Octadecenoic acid, trimethylsilyl ester. Isomer 2	Cac-16	0.179	0.252	0.210	0.139	0.624	7	11	0.676
Octadecenoic acid, trimethylsilyl ester. Isomer 3	Cac-17	0.366	2.133	0.641	0.006	0.350	1	5	0.657
Octadecenoic acid, trimethylsilyl ester. Isomer 4	Cac-18	0.095	0.436	0.168	0.000	0.420	0	2	0.153
Arachidonic acid, trimethylsilyl ester	Cac-19	0.061	0.193	0.058	0.065	0.624	3	2	0.173
Eicosenoic acid, trimethylsilyl ester. Isomer 1	Cac-20	0.019	0.101	0.029	0.007	0.907	1	1	0.039
Eicosanoic acid, trimethylsilyl ester	Cac-21	0.109	0.244	0.055	0.180	0.236	8	4	0.456
Docosanoic acid, trimethylsilyl ester	Cac-22	0.051	0.130	0.014	0.101	**0.047**	7	1	0.236
**Inorganic acid**									
Phosphoric acid, trimethylsilyl ester	–	0.275	1.474	–	–	–	4	6	1.015
**Nucleoside**									
Uridine, 2′,3′,5′-tris-O-TMS	–	0.066	0.154	–	–	–	3	7	0.362
**Steroids**									
Steroid Unidentified 1, trimethylsilyl derivative	Std-1	0.322	0.594	0.122	0.584	**0.047**	11	4	1.425
Steroid Unidentified 2, trimethylsilyl derivative	Std-2	0.010	0.030	0.000	0.023	0.107	4	0	0.088
Cholesterol trimethylsilyl ether	–	17.789	19.815	–	–	–	16	21	69.749
5α-Cholestan-3β-ol, trimethylsilyl derivative	Std-3	0.989	1.727	0.835	1.192	**0.028**	16	11	4.437
5α-Cholest-7-en-3β-ol, trimethylsilyl derivative	Std-4	0.230	0.490	0.225	0.236	0.591	6	12	0.788
Campesterol, trimethylsilyl ether	Std-5	0.431	0.708	0.652	0.141	**0.037**	6	19	1.545
β-Sitosterol, trimethylsilyl ether	Std-6	0.062	0.209	0.108	0.000	0.197	0	5	0.219
3-[(Trimethylsilyl)oxy]lanosta-9(11),24-diene	Std-7	0.656	1.710	0.803	0.464	0.591	8	7	1.784
**Sugar alcohols**									
D-Sorbitol, hexakis (trimethylsilyl) ether	–	0.001	0.005	–	–	–	1	1	0.013
Sugar alcohol 1, trimethylsilyl derivative	–	0.001	0.005	–	–	–	1	1	0.002

By far the most abundant class of compounds were steroids (mean of the summed relative amounts of steroids per sample = 20.49), followed by carboxylic acids (2.14), carbohydrates (1.51) alkanes (0.40) and alcohols (0.20). In addition, two amines (0.09), two sugar-alcohols (0.002), one inorganic acid (0.28) and one nucleoside (0.07) were also identified.

### Sexual variation in chemical composition

The number of compounds was similar between sexes (Wilcoxon rank sum test with continuity correction; *P* = 0.33; males: mean ± SD = 15.67 ± 8.36; females: mean ± SD = 13.06 ± 8.59). Males and females differed in the amounts of the main compound classes, with a tendency for males to have larger amounts of particular chemicals. However, this difference was only significant in the case of carbohydrates, for which males had statistically larger amounts (Wilcoxon rank sum test with continuity correction; *P* = 0.04; Fig. S2).

**Figure 5:**
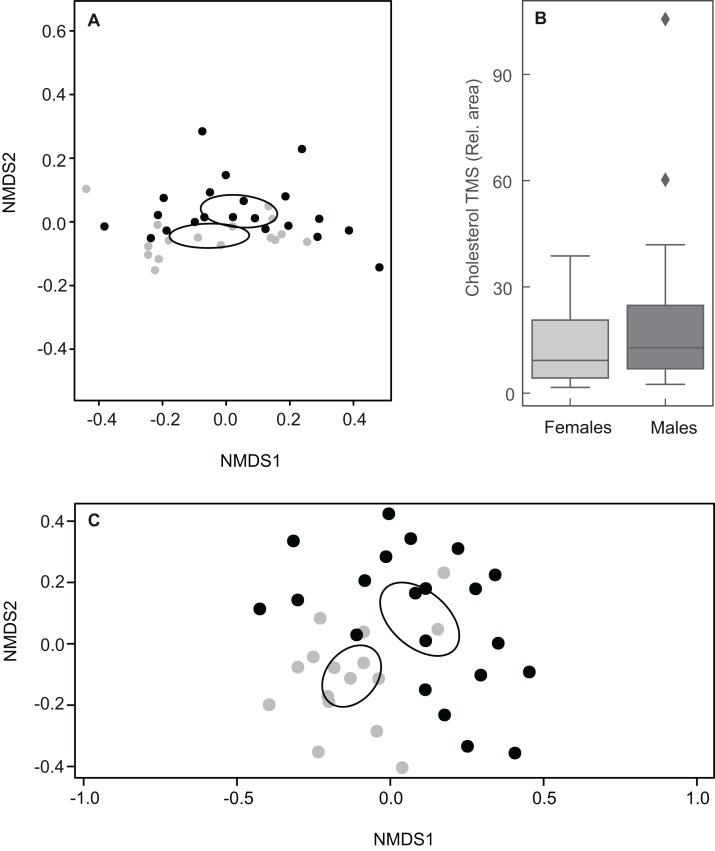
Plots showing sexual variation in chemical composition of mental glands of *Mauremys leprosa*. (A) Non-metric multidimensional scaling plots (NMDS) based on Bray Curtis dissimilarity considering all compounds (stress = 0.07). (B) Boxplot showing the amount (relative area) of cholesterol trimethylsilyl ether (TMS) in males and females. Median, interquartile range and outliers/extreme values are shown. (C) Non-metric multidimensional scaling plots (NMDS) based on Bray Curtis dissimilarity excluding cholesterol trimethylsilyl ether (stress = 0.21). In (A) and (C), gray points represent females and black points males. Closer points represent more similar compositions in individual turtles. Ellipses were calculated with the function *ordiellipse* (package Vegan) and represent 95% confidence interval (based on standard error) for the sexes.

Taking into account the relative abundances of compounds, the chemical composition of MG secretions did not differ clearly between males and females when including all 61 constituents (ANOSIM: *R* = 0.053, *P* = 0.095; Fig. 5A). The relative amount of cholesterol trimethylsilyl ether was highly variable among individuals, but amounts between sexes were similar (Fig. 5B). Indeed, a second NMDS plot excluding cholesterol showed a clear pattern discriminating between the sexes as revealed by different centroids and non-overlapping confidence intervals (ANOSIM: *R* = 0.31, *P* = 0.001; Fig. 5C).

**Figure 6:**
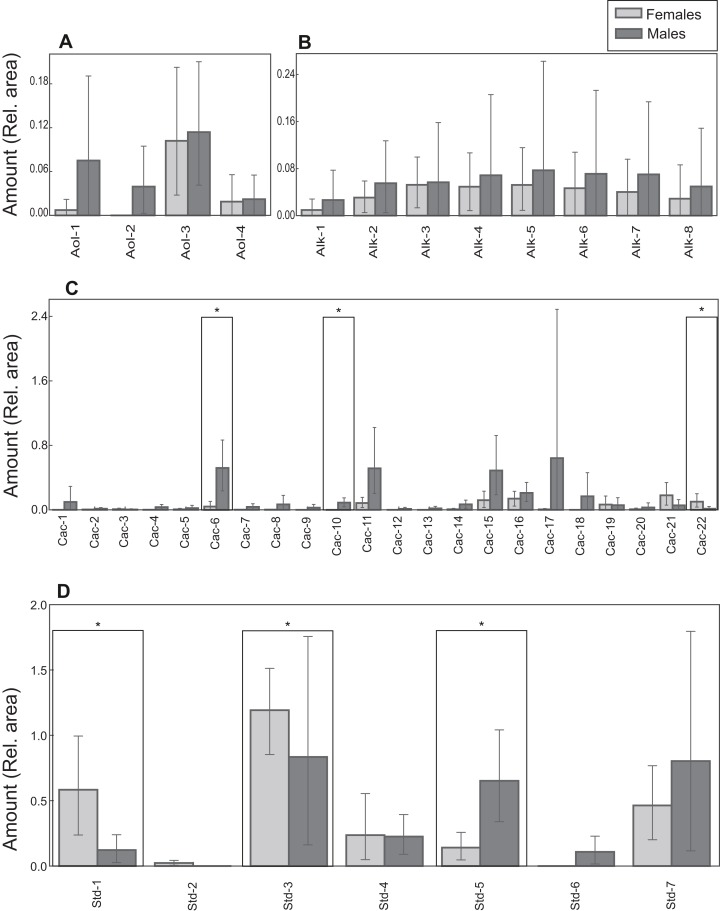
Chemical profiles of *M. leprosa*. Amount (mean and 0.95 confidence interval estimated by bootstrapping) of: (A) Alcohols (Aol). (B) Alkanes (Alk). (C) Carboxylic acids (Cac). (D) Steroids (Std; except cholesterol trimethylsilyl ether). Males are represented by black and females by gray color. Significant values after adjustment for multiple comparisons are marked with an asterisk. Compound abbreviations are as in Table 1.

Lipid profiles were examined through statistical tests for a further 41 compounds to detect potential differences between the sexes. Sexual differences occurred in three carboxylic acids and three steroids. Males had larger amounts of tetradecanoic acid trimethylsilyl ester, hexadecenoic acid trimethylsilyl ester (isomer 1) and campesterol trimethylsilyl ether (Table 1; Fig. 6). On the other hand, docosanoic acid trimethylsilyl ester, unidentified steroid 1 trimethylsilyl derivative and 5α-cholestan-3β-ol, trimethylsilyl derivative were significantly more abundant in females (Table 1; Fig. 6). Alcohol and alkane profiles were similar between males and females (Fig. 6). The chemical composition of male MG secretions showed a significant but weak difference between the seasons (i.e., sampling occasions; year 2018 vs year 2019) when considering all compounds, including cholesterol trimethylsilyl ether (ANOSIM: *R* = 0.176; *P* = 0.044). However, when cholesterol trimethylsilyl ether was excluded there was no clear seasonal pattern in chemical composition (ANOSIM: *R* = 0.141, *P* = 0.063; Fig. S3).

## Discussion

This study provides a comprehensive assessment of MG anatomy and chemistry in a freshwater turtle species. The MGs of adult *M. leprosa* consist of four independent sac-shaped invaginations opening in the gular region of the neck. Glands might differ slightly in size in a given individual but they show similar structure. In terms of general morphological complexity, the MGs of *M. leprosa* lie halfway between the simple invaginations present in emydid turtles and large, compound and elaborate glands of the desert tortoise *Gopherus agassizi* and the aquatic turtle *Siebenrockiella crassicollis* (Winokur & Legler, 1975). Histological examination showed that the glands of males are more complex than those present in females. In addition, chemical analysis revealed a relatively high diversity of compounds in MGs. Sexual differences in gland chemistry were driven by six of the compounds present in MGs, as well as a tendency for higher relative amounts in many other compounds in males. Three compounds were found in significantly larger amounts in males than in females and three others showed the reciprocal pattern.

### Chemical compounds in mental gland secretions

Steroids and carboxylic acids were the most common chemical compounds in MGs of the Spanish terrapin, a pattern also found in other reptiles (Weldon, Flachsbarth & Schulz, 2008; Martín & López, 2011). The most prevalent compound was cholesterol (trimethylsilyl ether), a steroid occurring in cell membranes of vertebrate tissues as well as in epidermal and glandular secretions of reptiles (Weldon, Flachsbarth & Schulz, 2008). The role of cholesterol in intraspecific signaling is unclear, with previous studies suggesting that it might be species-specific. One hypothesis is that cholesterol could act as an unreactive matrix protecting semiochemical functional compounds, found in smaller amounts, from accelerated degradation due to the effect of high temperatures in hot environments (Escobar et al., 2003). In the Spanish terrapin, we observed extremely high inter-individual variation in cholesterol trimethylsilyl ether that indeed masked differences between sexes in chemical compounds. Therefore, it is unlikely that cholesterol is directly involved in intraspecific communication in *M. leprosa*.

Previous studies on gopher tortoises (Rose, Drotman & Weaver, 1969; Rose, 1970) reported a different chemical composition of MG secretions compared to those of Spanish terrapins. Although distinctive chemical profiles of MG secretions could be expected due to the strongly divergent habitats occupied by both species, the different methods used in both studies (e.g., chemical analysis procedure and/or derivatization agents) render the comparison preliminary. Nonetheless, some interesting patterns emerge. First, cholesterol and saturated and unsaturated fatty acids in the range of C_8_–C_18_ carbon chain lengths have been identified in secretions of *Gopherus berlandieri*, whereas MGs of *M. leprosa* contained saturated and unsaturated C_8_–C_20_ carboxylic acids, as well as shorter compounds such as benzoic and propanoic acids (the latter occurring only in two males). Therefore, Spanish terrapin secretions encompass a larger chain length range of carboxylic acids than those of *G. berlandieri*. The xeric terrestrial environment inhabited by *G. berlandieri* might explain the lack of highly volatile compounds such as relatively short chain carboxylic acids that would fade out quickly due to the degradative effect of elevated temperatures (Alberts, 1992; Van Oudenhove et al., 2011; Martín & López, 2013). In contrast, Spanish terrapins are predominantly aquatic—but may also be active on land (see below)—and therefore occupy habitats associated with more mesic conditions than *Gopherus*, where more volatile compounds such as relatively short carboxylic acids (e.g., benzoic and propanoic acids) could persist for a longer time. Second, some compounds present in Spanish terrapin MGs such as alcohols, alkanes and carbohydrates have not been reported in *G. berlandieri* (Rose, 1970). However, it is important to point out that methodological differences between the studies could affect the results and therefore a comparision between our findings and the previous studies needs to be considered carefully. Compounds with relatively high polarity, such as carbohydrates, can solve better in water and thus facilitate the reception of other compounds that might be chemosignals from glandular secretions by turtles that are actively mating. These compounds are potentially important since copulations in *M. leprosa* typically, but not exclusively, occur underwater (Bertolero & Busack, 2017) and experiments indicate that turtles might use water-borne chemical cues to communicate with other conspecifics (Ibáñez, López & Martín, 2012; Ibáñez et al., 2013).

### Sexual dimorphism in mental gland anatomy and chemistry

MGs differ clearly in terms of anatomical and structural complexity between males and females. In males, the orifice of the gland is followed by a simple duct connected to the lumen in which secretion accumulates. Secretions had a positive (weak) reaction to PAS indicating the presence of carbohydrates and/or neutral mucosubstances, in line with *Gopherus* males, in which highly vacuolated cells present in MGs showed a PAS-positive reaction (Winokur & Legler, 1975). The presence of well-developed lipid droplets was not observed in *M. leprosa* glands. However, electron-light bodies are present in the lumen of the gland but the chemical nature of these remains unknown. We detected a relatively high degree of exocytotic vacuolization in mature cells that disintegrate in the lumen, indicating that MGs in male *M. leprosa* are active and produce holocrine secretions composed of cellular and organellar fragments as well as exocytotic products.

MGs consist of heavily keratinized invaginations in females. Histological evidence in *Gopherus berlandieri* showed that MGs are active in both sexes (Weaver, 1970). However, inactive glands have been described in females and juveniles of other chelonians, including species of the family Geoemydidae (Winokur & Legler, 1975). Similarly, the histology and fine structure of MGs in female *M. leprosa* suggest that they might be inactive or at least reduced. No evidence of holocrine secretion in female glands was observed in this study. Female glands are likely vestigial or primordium organs in *M. leprosa*.

An interesting feature of MG chemistry in Spanish terrapins is the presence of carbohydrates, found in larger amounts in males than in females. One explanation is that sugars found in MGs are involved in glycosylation of secreted proteins, a process affecting the three dimensional conformation of the molecules and therefore their function (Moremen, Tiemeyer & Nairn, 2012). Although the protein fraction of chelonian MGs is nearly unknown (Alberts, Rostal & Lance, 1994), proteins could function as potential signals (Wyatt, 2014). It is tempting to speculate that the sugars found in MG secretions of *M. leprosa* are involved in the glycosylation and regulation of sexually-mediating glycoproteins expressed in male glands. Proteomic analysis of MGs should shed light on this hypothesis.

Some compounds appeared in larger amounts in one of the sexes and deserve special attention as they could potentially be involved in chemical signaling. Males had relatively larger amounts of campesterol trimethylsilyl ether, a steroid, as well as hexadecenoic acid trimethylsilyl ester (isomer 1) and tetradecanoic acid trimethylsilyl ester, both of which are carboxylic acids. A possible explanation is that male terrapins allocate these compounds from their fat stores to the MGs, where they accumulate in the secretions until being released. However, most of the research on chemical communication in aquatic or semiaquatic turtles focuses on the freshwater environment (Muñoz, 2004; Poschadel, Meyer-Lucht & Plath, 2006; Lewis et al., 2007; Ibáñez, López & Martín, 2012) and it is unclear how carboxylic acids could be transmitted in this media. Spanish terrapins might also be able to communicate in the terrestrial environment as they spend long periods basking (Díaz-Paniagua, Andreu & Keller, 2015; Bertolero & Busack, 2017). Carboxylic acids might be used as olfactory signals on land for mating and/or intrasexual interactions as postulated for *G. berlandieri* (Rose, 1970). This could be important in male–male interactions during basking site competition, as optimal sites for thermoregulation are a limiting factor in natural habitats (Cadi & Joly, 2003; Polo-Cavia, López & Martín, 2010). Campesterol is a phytosterol present in the epidermis and femoral secretions of squamates (Weldon, Flachsbarth & Schulz, 2008). Phytosterols are typically of plant origin and given that *M. leprosa* is omnivorous, campesterol could be obtained from the intake of plant material. Vertebrates might excrete steroids that serve as olfactory cues involved in social communication (Doyle & Meeks, 2018). In goldfish, steroid-derived pheromones are released into the water to modulate reproductive behavior by synchronizing female–male cycles (Dulka et al., 1987). However, the potential mechanism of action of these compounds in turtles is unknown. In *M. leprosa*, chemosignal detection is dependent on concentration (Ibáñez et al., 2014). However, without a behavioral bioassay or a physiological experiment, we cannot establish whether these compounds are involved in sexual signaling. Nonethless, our study provides the groundwork for future empirical assessments of the roles of these compounds in chemical communication in turtles.

Two steroids (5α-cholestan-3β-ol trimethylsilyl derivative and an unidentified steroid) and one carboxylic acid (docosanoic acid trimethylsilyl ester) were found in larger amounts in female glands. We hypothesize that these come from metabolism of other compounds and/or are present in large amounts in turtle skin. In fact, 5α-cholestan-3β-ol can be metabolically converted from cholesterol (Shefer, Milch & Mosbach, 1964; Werbin, Chaikoff & Phillips, 1964). A plausible explanation is that chemical compounds found in female glands originate from the excess of accumulated keratin in corneocytes, as carboxylic acids—especially very long chain (C_20_–C_28_) ones—and cholesterol among other compounds are found in abundance in these cells (Downing, 1992). However, we cannot rule out that these compounds are used by females to signal their quality to potential partners (Ibáñez, López & Martín, 2012), although the simple structure of MGs in females and a lack of glandular epithelium argues against it. It is also possible that chemical signals in turtles are produced by other kinds of secretory organs such as cloacae and/or Rathke’s glands.

We note that other compounds might also be important in sexual communication but could be overlooked or masked by the high level of inter-individual variability in chemical composition shown by Spanish terrapins. In general, and in line with the rudimentary character of female MGs, males tended to have a higher compound diversity and relatively elevated amounts of most compounds. Several compounds were present in males but were entirely absent in females. However, only one compound was absent in males but was present in some females (unidentified steroid 2, trimethylsilyl derivative; see Table 1). In many instances, these “exclusive” compounds were found in only a small number of males. Therefore, it could be that other factors such as age, body size or health status of male turtles influence the chemical composition of MGs.

Slight seasonal differences in chemical composition were observed in male turtles but this was mostly driven by variable levels of the main compound—cholesterol, and therefore we conclude that there is no clear pattern of temporal variation in chemical composition of MG secretions. MG size (volume) in male *G. agassizii* changes through the year being larger during the mating season (Alberts, Rostal & Lance, 1994). Although our results in male *M. leprosa* do not show a clear difference between seasons, our sampling occasions, August–September 2018 and March 2019, might have encompassed reproductive periods in this species (Díaz-Paniagua, Andreu & Keller, 2015), and thus were not designed to discern seasonal variation in semiochemical production.

## Conclusions

This study showed that MGs in male *M. leprosa* are complex structures producing holocrine secretions. In contrast, female MGs resemble inactive or rudimentary organs. The bulk of the MG secretion is composed of steroids, especially cholesterol, as in other reptilian epidermal glands. Males and females showed qualitatively similar types of compounds in MGs, suggesting that many chemicals could be metabolic byproducts excreted from skin cells with no obvious role as sexual signals. However, the relative amounts of some compounds were higher in males, with significantly larger amounts of three compounds. Moreover, significant amounts of carbohydrates were found in glandular secretions, especially in males; their role is unknown but they could be important in protein glycosylation. Through biossays, behavioral or physiological experiments, future research should test whether any of the compounds identified in this study play a role in intraspecific communication. In parallel, studies on the protein fraction should be done to identify proteins used in chemosignalling. Furthermore, a uniform chemical identification method applied to MG secretions from a wider set of turtle species could address the effect of environment in shaping interspecific variation in chelonian chemical signals.

## Supplemental Information

10.7717/peerj.9047/supp-1 Supplemental Information 1 Sexual variation in mental gland composition of *M. leprosa*, based on the percentages of the compounds. Non-metric multidimensional scaling (NMDS) plot based on Bray Curtis dissimilarity. A) NMDS plot considering all compounds (stress = 0.098). B) NMDS plot excluding cholesterol trimethylsilyl ether (stress = 0.17). In this analysis percentages were used instead of the relative areas in respect to the internal standard (see “METHODS”). Closer points represent more similar compositions in individual turtles. Ellipses were calculated with the function ordiellipse (package Vegan) and represent 95% confidence interval (based on standard error) for the sexes.

10.7717/peerj.9047/supp-2 Supplemental Information 2 Chemical compound classes in male (m) and female (f) mental gland secretions of *M. leprosa*. Mean ± 95% CI (estimated by bootstrapping) for the relative areas of the distinct classes are shown. Steroids include cholesterol. Others include an inorganic acid, a nucleoside, two amines and two sugar alcohols.

10.7717/peerj.9047/supp-3 Supplemental Information 3 Seasonal (inter-year) variation in mental gland composition in males of *M. leprosa*, based on the relative areas of the compounds. Non- metric multidimensional scaling (NMDS) plot calculated from Bray Curtis dissimilarity among samples and compounds (excluding cholesterol). Blue points represent year 2018, and red points represent 2019. Closer points represent more similar compositions in individual turtles. Only males were considered for this analysis. Ellipses were calculated with the function ordiellipse (package Vegan) and represent 95% confidence interval (based on standard error) for the years. Stress = 0.13

## Additional Information and Declarations

### Data Availability

The following information was supplied regarding data availability: The raw data is available at the Jagiellonian University Repository (RUJ): - Raw images for histology pictures of the article: “The chemistry and histology of sexually dimorphic mental glands in the freshwater turtle, *Mauremys leprosa*”: https://ruj.uj.edu.pl/xmlui/handle/item/152495. - RAW chromatographic data (GC-MS) for the article: “The chemistry and histology of sexually dimorphic mental glands in the freshwater turtle, *Mauremys leprosa*”: https://ruj.uj.edu.pl/xmlui/handle/item/152541. - Research datasets of the article: “The chemistry and histology of sexually dimorphic mental glands in the freshwater turtle, *Mauremys leprosa*”: https://ruj.uj.edu.pl/xmlui/handle/item/152499.
