# Supplementary figures and images for: The chemistry and histology of sexually dimorphic mental glands in the freshwater turtle, Mauremys leprosa
Source: PeerJ. 2020 May 15;8:e9047. doi: 10.7717/peerj.9047 (PMC7233278; doi:10.7717/peerj.9047)

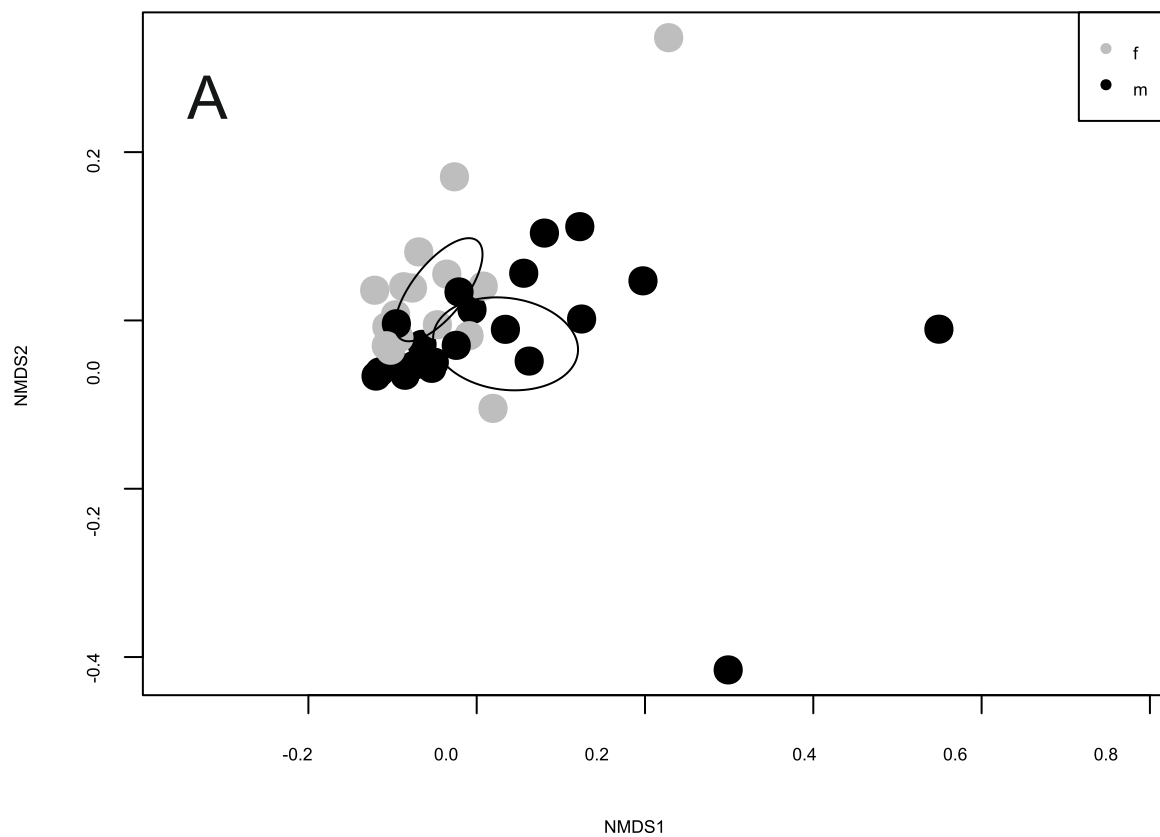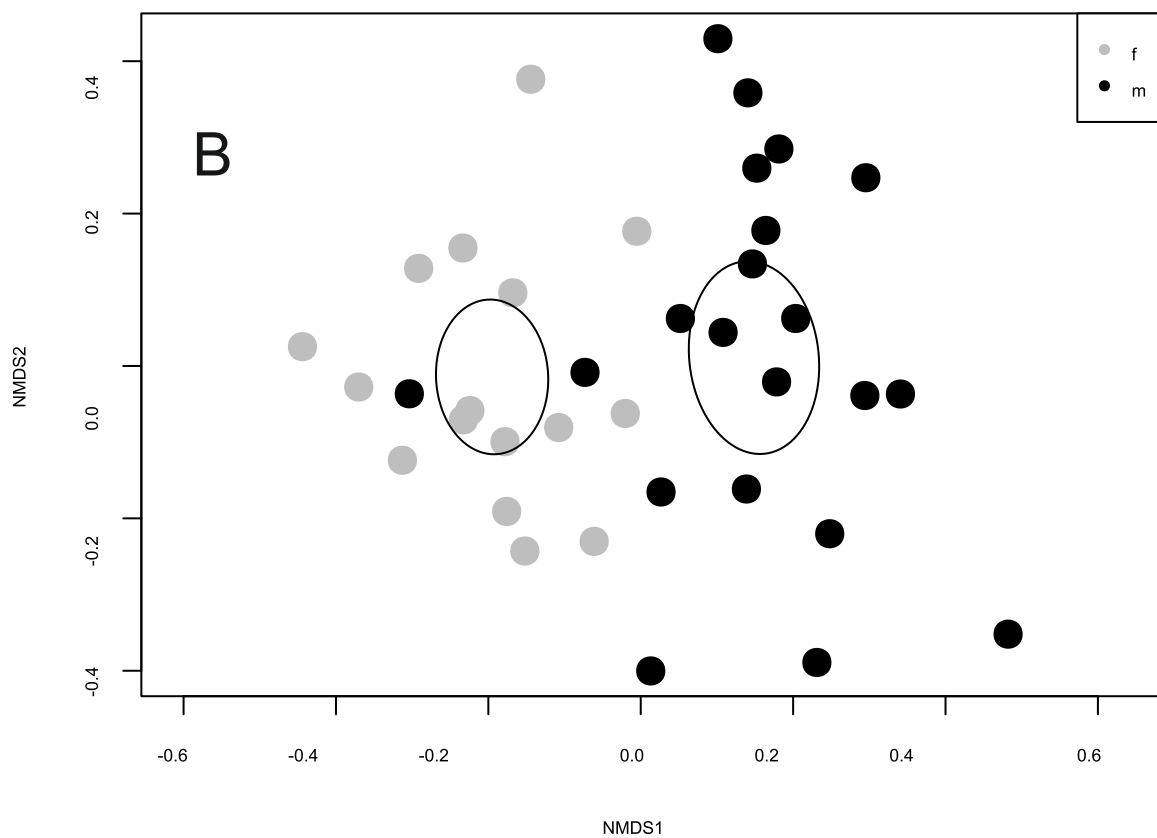

Supplement: Supplemental Information 1 — Non-metric multidimensional scaling (NMDS) plot based on Bray Curtis dissimilarity. A) NMDS plot considering all compounds (stress = 0.098). B) NMDS plot excluding cholesterol trimethylsilyl ether (stress = 0.17). In this analysis percentages were used instead of the relative areas in respect to the internal standard (see “METHODS”). Closer points represent more similar compositions in individual turtles. Ellipses were calculated with the function ordiellipse (package Vegan) and represent 95% confidence interval (based on standard error) for the sexes. [file peerj-08-9047-s001.pdf]

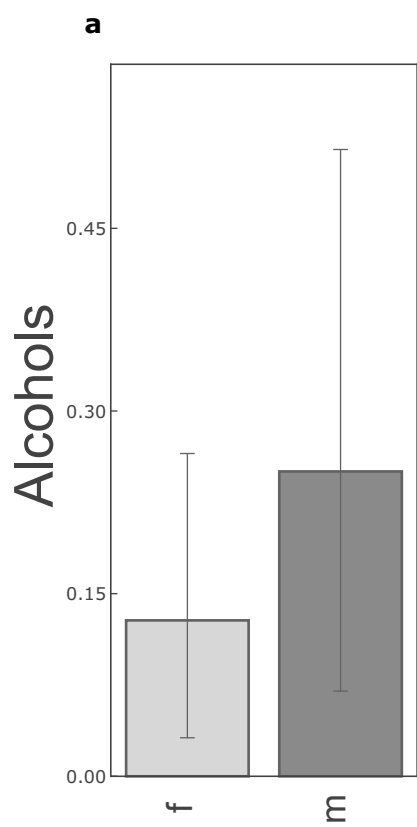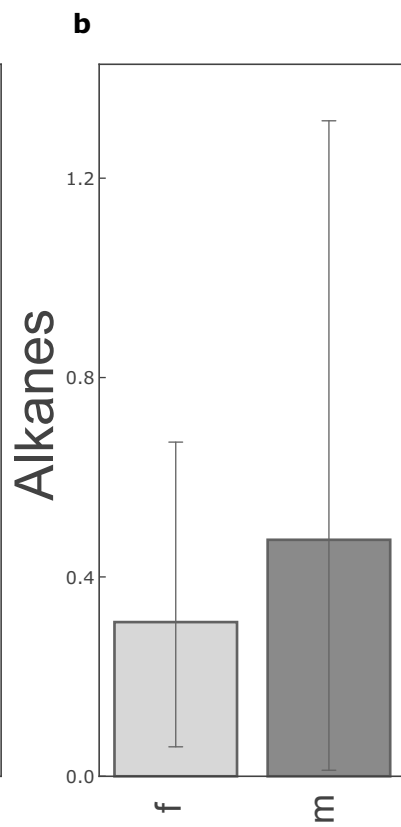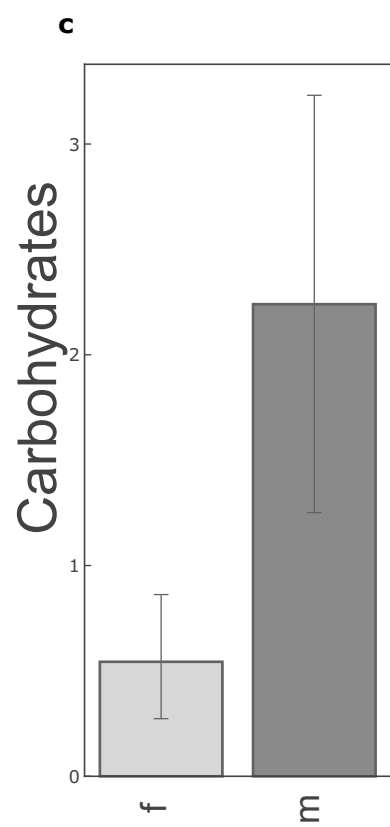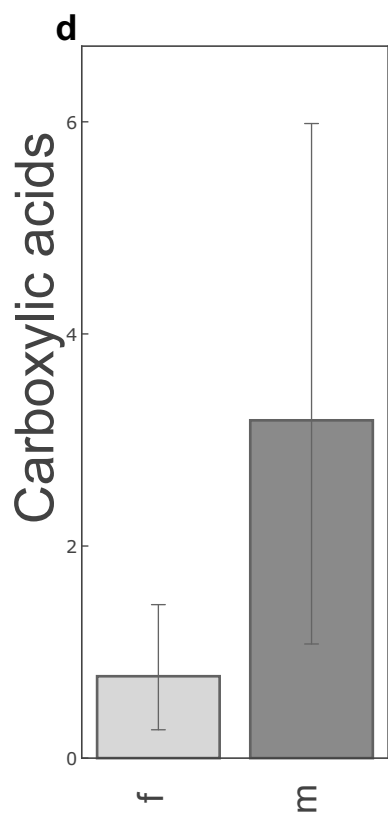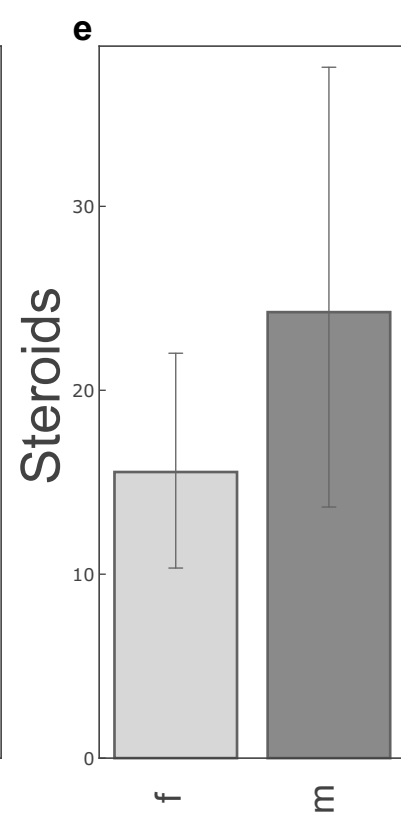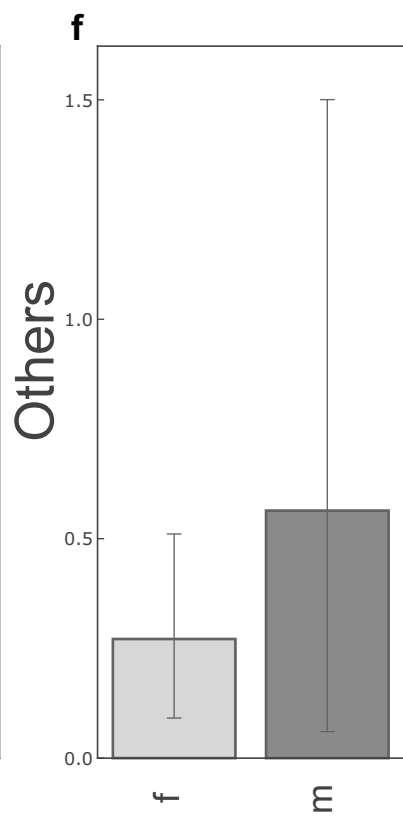

Supplement: Supplemental Information 2 — Mean ± 95% CI (estimated by bootstrapping) for the relative areas of the distinct classes are shown. Steroids include cholesterol. Others include an inorganic acid, a nucleoside, two amines and two sugar alcohols. [file peerj-08-9047-s002.pdf]

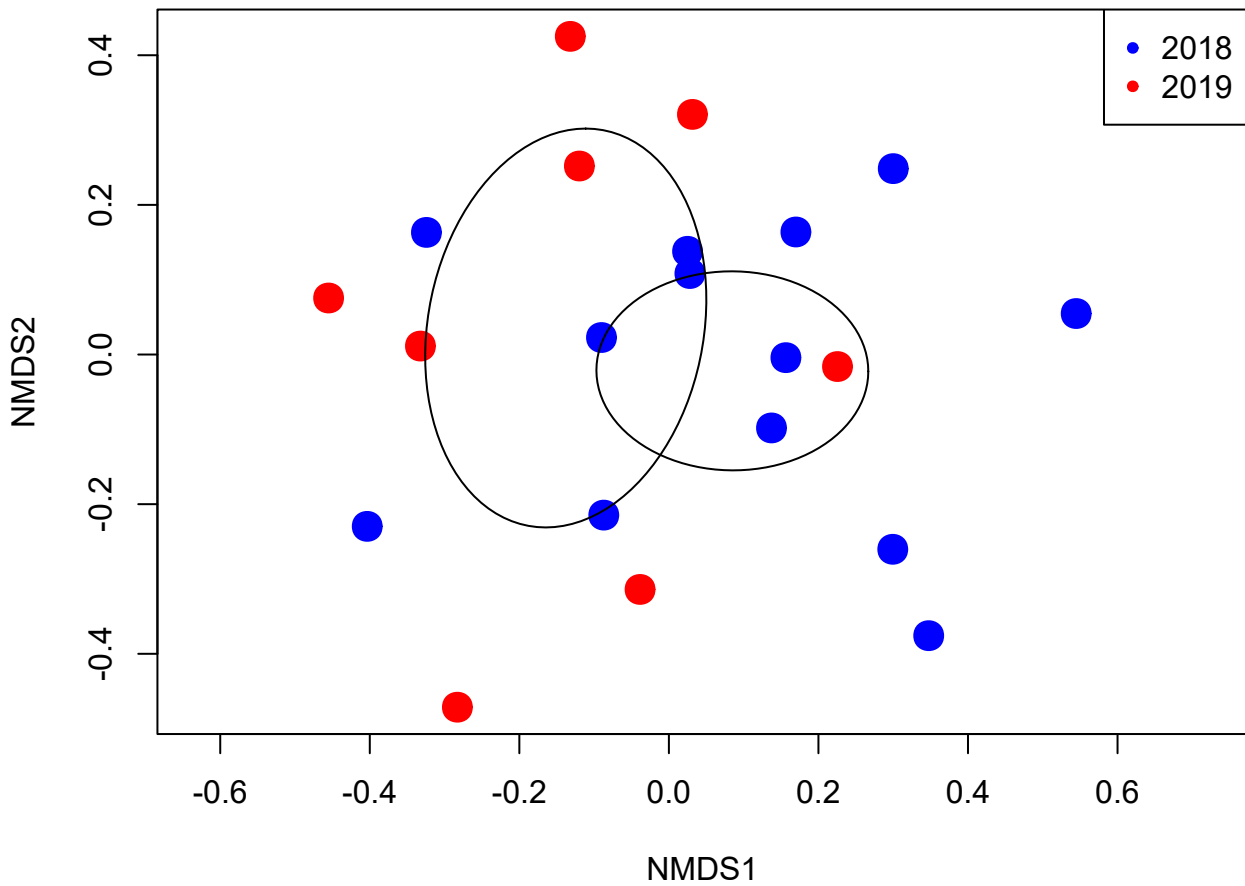

Supplement: Supplemental Information 3 — Non- metric multidimensional scaling (NMDS) plot calculated from Bray Curtis dissimilarity among samples and compounds (excluding cholesterol). Blue points represent year 2018, and red points represent 2019. Closer points represent more similar compositions in individual turtles. Only males were considered for this analysis. Ellipses were calculated with the function ordiellipse (package Vegan) and represent 95% confidence interval (based on standard error) for the years. Stress = 0.13 [file peerj-08-9047-s003.pdf]
